# Accidental Dural Puncture in Obstetrics: A Headache to the Anesthesiologist

**DOI:** 10.7759/cureus.94866

**Published:** 2025-10-18

**Authors:** Marta Marques, Sara Matos, Catarina Sampaio

**Affiliations:** 1 Anesthesiology, Unidade Local de Saúde São João, Porto, PRT; 2 Anesthesiology, Unidade Local de Saúde Alentejo Central, Évora, PRT

**Keywords:** accidental dural puncture, complication of neuraxial anesthesia, epidural blood patch, obstetric anesthesia and analgesia, post‑dural puncture headache

## Abstract

Post-dural puncture headache (PDPH) is a known complication of neuraxial techniques. Pregnant women are particularly predisposed to this condition. When it occurs, it can increase hospital costs due to prolonged stays and adversely affect maternal well-being, delivery experience, and newborn care.

This case report underscores the importance of considering differential diagnoses for postpartum headache, including complications related to neuraxial procedures such as epidural catheter placement and epidural blood patch (EBP).

Although PDPH is a well-recognized complication, special attention is warranted in cases where symptoms persist despite initial treatment, including the need for a second EBP. Moreover, rare but serious complications such as meningitis should always be considered, as they can significantly delay maternal recovery, extend hospitalization, and interfere with both newborn care and the overall postpartum experience.

## Introduction

Accidental dural puncture during epidural catheter placement is one of the most concerning complications of neuraxial techniques among obstetric anesthesiologists. Its estimated incidence ranges from 0.5% to 1.5% in women in labor [1-3]. Post-dural puncture headache (PDPH) is a common adverse outcome of this complication, occurring in approximately 50%-80% of postpartum patients and typically presenting within the first five days following dural puncture [3-6]. Risk factors for PDPH include young age, a history of headaches, the use of cutting needles, and wider-gauge needles [3,4]. The most widely accepted mechanism is cerebrospinal fluid (CSF) hypotension resulting from CSF leakage [3]. PDPH is often described as a dull, throbbing pain in the frontal or occipital region that worsens when sitting or standing and improves when lying down [3-7]. Additional symptoms may include neck stiffness (10%-25%), nausea and vomiting (20%), auditory symptoms such as tinnitus (10%), visual disturbances like diplopia (2%) and photophobia (23%), and, rarely, cranial nerve palsies involving the facial or abducens nerves (<1%) [3,4]. The headache typically resolves within two weeks or after closure of the dural puncture [4]. Headache affects nearly one-third of postpartum women, underscoring the importance of a thorough differential diagnosis to identify both common and potentially serious causes [7-11]. Clinically significant differentials include meningitis, cerebral ischemia, intracranial hemorrhage, neurotoxicity, pneumocephalus, cerebral venous thrombosis, caffeine withdrawal, and tension headache. Additionally, complications associated with dural puncture and CSF leakage, such as subdural hematoma and cerebral venous sinus thrombosis, can result in severe headache [3,7,12,13]. Therefore, determining the etiology, management, and treatment of headaches in postpartum patients can be particularly challenging for anesthesiologists, especially when the clinical course is atypical or complex [4,12-14].

## Case presentation

A 29-year-old primigravida at 39 weeks and 2 days of gestation, classified as American Society of Anesthesiologists Physical Status (ASA-PS) II, with a history of smoking and tension headaches, was admitted to the labor ward for epidural catheter placement for labor analgesia. The risks associated with the procedure were explained, and informed consent was obtained.

During the initial attempt to place the epidural catheter at the L2-L3 interspace, using an 18-gauge Tuohy needle and the loss-of-resistance-to-saline technique under sterile conditions, an accidental dural puncture occurred, evidenced by the visualization of CSF at the needle hub. The needle was removed, the patient repositioned, and the procedure was repeated at the L3-L4 level, where the epidural catheter was successfully placed without complications. Throughout labor, the patient was continuously monitored for oxygen saturation, heart rate, non-invasive blood pressure, and cardiotocography. All bolus administrations were performed by an anesthesiologist, with continuous observation for signs or symptoms of a possible subarachnoid block, given the potential risk of epidural catheter migration into the subarachnoid space through the dural laceration or direct drug leakage via the puncture site. Vaginal delivery occurred approximately 11 hours after hospital admission without complications, and the epidural catheter was removed following placental expulsion.

On post-dural puncture day 1, the patient developed a headache that worsened when sitting or standing, accompanied by neck stiffness and auditory tinnitus. She exhibited no other neurological signs and remained afebrile. The following management measures were implemented: patient education on warning signs, encouragement of oral hydration, intake of 2-3 caffeinated beverages within 24 hours, administration of analgesics as needed, and restriction of ambulation or upright positioning during symptomatic periods.

On day 2, due to worsening clinical symptoms, specifically persistent severe orthostatic headache, neck pain, tinnitus, and the development of photophobia, the patient was offered an epidural blood patch (EBP).

The risks of the procedure were explained to the patient, who demonstrated understanding and provided consent. She was then admitted to the operating room, where the procedure was performed under aseptic conditions. An EBP was administered at the L2-L3 interspace and discontinued after the injection of 8 mL of autologous blood due to the onset of severe lower back pain. The patient remained in a supine position for two hours in the recovery room before being transferred to the inpatient unit. Close clinical monitoring and supportive measures were continued until day 3; however, no improvement in symptoms was observed following the EBP.

On day 3, due to persistent severe symptoms without clinical improvement, the treatment plan was reassessed, a scheduled analgesic regimen was initiated, and a Neurology consultation was requested for comprehensive evaluation. The neurological examination revealed no focal deficits.

On day 4, the patient continued to experience severe symptoms, prompting a contrast-enhanced CT scan to rule out differential diagnoses, particularly cerebral venous sinus thrombosis. Imaging revealed slight pituitary enlargement but no other significant abnormalities.

On day 5, due to persistent severe orthostatic headache, neck pain, photophobia, and tinnitus, and in the absence of neurological signs or fever, a repeat EBP was proposed, to which the patient consented. The second EBP was performed in the operating room under aseptic conditions at the L3-L4 level, with 20 mL of autologous blood administered without complications. The patient remained in a supine position for two hours in the recovery room before being transferred to the inpatient unit, where she received as-needed analgesia. The patient reported complete symptom resolution following the procedure. Approximately three hours after the EBP, and after discontinuation of scheduled analgesia, the patient developed an isolated febrile episode of 38.9 °C. The Infectious Diseases team was consulted, and blood cultures, urine cultures, urinary antigen tests, and a lumbar CT scan were requested. Lumbar puncture was not performed due to the history of severe PDPH and the two prior EBPs. Despite no abnormalities or collections being detected on lumbar CT, empirical antibiotic therapy with vancomycin and meropenem was initiated for suspected nosocomial meningitis, considering the multiple neuraxial procedures and a clinical picture including headache, neck stiffness, photophobia, and fever. During vancomycin infusion, the patient developed "red man syndrome" with rash, pruritus, and hypotension, which resolved promptly after reducing the infusion rate. The Obstetrics and Gynecology team also noted breast engorgement and erythema, another potential cause of the fever. To prevent antibiotic exposure to the neonate, breastfeeding was temporarily discontinued.

On day 6, the patient experienced complete resolution of symptoms. Results from aerobic and anaerobic blood cultures, fungal cultures, urine cultures, and urinary *Streptococcus pneumoniae* antigen tests were all negative. In a multidisciplinary discussion, given the inability to confirm meningitis through CSF analysis, the decision was made to continue empirical intravenous antibiotic therapy. The patient remained hospitalized until post-dural puncture day 12 and completed the remainder of the antibiotic course through a home hospitalization program until day 22. During this period, she remained asymptomatic.

## Discussion

Evidence-based management of PDPH

One-third of postpartum women experience headache, 75% of which are primary [1,3]. In this case, the confirmed CSF leakage secondary to accidental dural puncture established CSF hypotension as the primary etiological factor. Since PDPH is a clinical diagnosis, the patient was immediately flagged for reassessment and management in line with the latest guidelines [4].

These guidelines recommend the following: no prophylactic restriction of movement or upright positioning, as bed rest is no longer advised except for temporary symptomatic relief; avoidance of prophylactic fixed analgesic regimens, while prioritizing multimodal analgesia (paracetamol, nonsteroidal anti-inflammatory drugs, and mild opioids); encouragement of oral fluids to promote normohydration, with intravenous fluids if required; and the use of caffeine within the first 24 hours of headache (up to 300 mg/day if breastfeeding, and up to 900 mg/day otherwise) [3-7].

The management of the present case followed these recommendations; however, non-invasive measures proved insufficient [4]. Approximately 48 hours following dural puncture, an EBP was proposed and performed under aseptic conditions in the operating room. Administration of autologous blood was interrupted at 8 mL due to severe lumbar pain. Given the persistence and worsening of headache, neck stiffness, and photophobia, other differential diagnoses were considered [8-13].

Interpretation of refractory symptoms

Clinical re-evaluation, including neurological examination and contrast-enhanced CT, was performed to exclude complications such as subdural hematoma, cavernous sinus thrombosis, and reversible posterior encephalopathy, conditions that can arise as sequelae of CSF hypotension. Septic, aseptic, or drug-induced meningitis was also considered due to overlapping symptoms. The neurological exam showed no focal deficits, the CT revealed only slight pituitary engorgement, and the patient remained afebrile, with symptoms still consistent with severe PDPH. The most likely diagnosis was inefficacy of the first EBP.

The success rate of EBP has been shown to vary widely in recent studies, ranging from 33% to 91% [4]. Since the patient did not tolerate more than 8 mL of autologous blood during the first attempt, the medical team considered the possibility of technical failure. If symptoms persist without change in nature and no alternative diagnosis is supported clinically or radiologically, repetition of the EBP is reasonable. Approximately 15% of patients with PDPH require at least two EBPs for sustained symptom resolution [4,7,14].

Risk-benefit of invasive testing following EBP

Concerns arose when the patient developed a fever spike a few hours after the second EBP and after discontinuation of the fixed analgesic regimen, which included paracetamol and ketorolac (both with antipyretic properties). This raised suspicion of overlapping meningitis, consistent with her clinical symptoms of headache, photophobia, neck stiffness, and fever [13].

The Infectious Diseases team recommended a lumbar puncture to exclude meningitis. However, the Anesthesiology team opposed this approach, citing the risk of recurrent PDPH given the prior dural puncture with documented CSF leakage and two EBPs already performed, the first being ineffective and the second apparently successful. Moreover, they emphasized concerns regarding the cutting needle and gauge required for lumbar puncture, as well as the potential difficulty in interpreting results due to autologous blood contamination after EBP and dural exposure.

Multidisciplinary management decisions

After multidisciplinary discussion, a prophylactic regimen for bacterial meningitis was initiated with intravenous vancomycin and meropenem to cover both nosocomial and community-acquired pathogens. Before starting antibiotics, samples were collected to exclude other infectious foci, all of which returned negative. The patient remained afebrile in the following days.

The Obstetrics and Gynecology team also noted breast engorgement and pain, coinciding with the initiation of antibiotic therapy, which resolved within approximately one day. This raised the possibility that mastitis could have contributed to the fever spike. However, given the severity of potential alternative diagnoses, the team elected to maintain prophylactic treatment for bacterial meningitis.

## Conclusions

This case illustrates the multifaceted challenges posed by accidental dural puncture in the obstetric population. Management required escalation from guideline-based conservative measures to invasive neuraxial techniques and ultimately raised concern for a possible neuroaxis infection, prompting broad-spectrum intravenous antibiotic therapy. These interventions contributed to prolonged hospitalization and a temporary interruption of exclusive breastfeeding. Although maternal symptoms resolved within five days of the initial epidural catheter placement and adverse drug reactions were promptly managed, the episode underscores not only the physical burden of PDPH but also its potential psychological impact on the mother, warranting follow-up.

Clinically, this report highlights three key learning points: (1) evidence-based management of PDPH: conservative measures, escalation to EBP, and recognition of variable efficacy; (2) interpretation of refractory symptoms: careful exclusion of differential diagnoses including neuroaxis complications and meningitis; and (3) multidisciplinary decision-making: balancing risks and benefits of invasive testing following EBP and coordinating care across specialties. By emphasizing these aspects, the case contributes to the understanding of how guideline-driven strategies intersect with real-world complexities in postpartum care.
